# The Use of Transcriptomics to Unveil the Role of Nutrients in Mammalian Liver

**DOI:** 10.5402/2013/403792

**Published:** 2013-08-28

**Authors:** Jesús Osada

**Affiliations:** ^1^Departamento de Bioquímica y Biología Molecular y Celular, Facultad de Veterinaria, Instituto de Investigación Sanitaria de Aragón, Universidad de Zaragoza, 50013 Zaragoza, Spain; ^2^CIBER de Fisiopatología de la Obesidad y Nutrición, Instituto de Salud Carlos III, 28029 Madrid, Spain

## Abstract

Liver is the organ primarily responding to diet, and it is crucial in determining plasma carbohydrate, protein, and lipid levels. In addition, it is mainly responsible for transformation of xenobiotics. For these reasons, it has been a target of transcriptomic analyses. In this review, we have covered the works dealing with the response of mammalian liver to different nutritional stimuli such as fasting/feeding, caloric restriction, dietary carbohydrate, cholesterol, fat, protein, bile acid, salt, vitamin, and oligoelement contents. Quality of fats or proteins has been equally addressed, and has the influence of minor dietary components. Other compounds, not purely nutritional as those represented by alcohol and food additives, have been included due to their relevance in processed food. The influence has been studied not only on mRNA but also on miRNA. The wide scope of the technology clearly reflects that any simple intervention has profound changes in many metabolic parameters and that there is a synergy in response when more compounds are included in the intervention. Standardized arrays to systematically test the same genes in all studies and analyzing data to establish patterns of response are required, particularly for RNA sequencing. Moreover, RNA is a valuable, easy-screening ally but always requires further confirmation.

## 1. Introduction

The postgenomic era poses a new challenge: to use genomic structural information, to display and analyze biological processes on a genome-wide scale, to assign gene function, and to know its response to different environmental stimuli. All functional genomic approaches such as genome-wide association studies, whole-genome and whole-exome sequencing, array-based comparative genomic hybridization, global DNA methylome mapping, and gene or noncoding RNA expression profiling [1] and other systems biology tools will be required to achieve such endeavor. 

Transcriptomic analyses cover the step of passing information from DNA to RNA. In contrast to DNA, there is not a single transcriptome but one for each cell. In addition, it may change in different circumstances. 

DNA microarrays are a miniaturized, ordered arrangement of nucleic acid fragments from individual genes located at defined positions on a solid support. They are powerful tools to detect changes in the expression of thousands of genes simultaneously by specific hybridization in multiple samples in parallel and identify the effect of different nutrients.

Recently, whole genome sequencing technologies have emerged as new high-throughput methods to quantify gene expression, epigenetic modifications, and DNA-protein binding. This approach provides a new perspective on the samples under study and complements microarray gene expression data.

This review focuses on the effect(s) of dietary interventions at the global level of gene expression determined by transcriptomic data mainly generated using cDNA microarrays. It would represent one aspect of the nutrigenomic approach considering this as the study of how dietary components interact with genes and their products to alter phenotype, and conversely how genes and their products metabolize these constituents into nutrients, antinutrients, and bioactive compounds. The ultimate goal of these studies would be the possibility of delivering personalized nutrition [2]. The focus is on the mammalian liver as the organ primarily responding to diet, being crucial in determining plasma carbohydrate, protein and lipid levels. In addition, it is responsible for a great deal of metabolization of xenobiotics. Most of studies have analyzed the organ without taking into consideration the heterogeneity of its cells and that some interventions may influence them. A more sophisticated approach such as the use of laser microdissection microscopy will be required as well as analyses of changes in isolated cell preparations [3].

**Figure 1:**
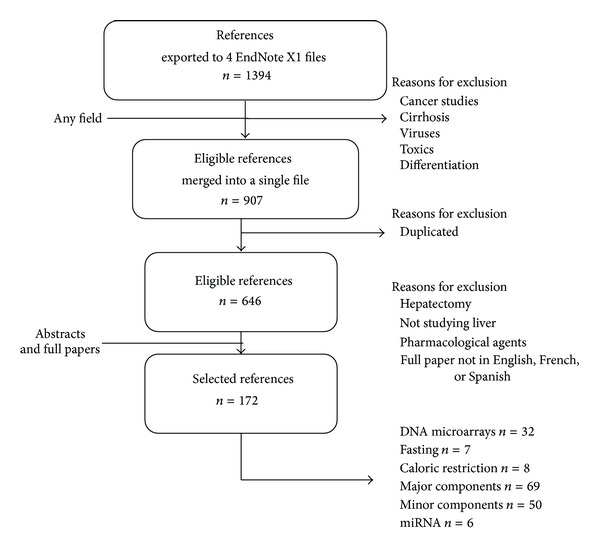
Flow chart displaying the stages used to select the references considered. EndNote X1 (Thomson Reuters, New York, NY, USA).

The present report has tried to adhere to systematic review guidelines [4]. As displayed in Figure 1, a search in PubMed (http://www.ncbi.nlm.nih.gov/pubmed/) using certain keywords (DNA microarray and liver, microarray and nutrient, transcriptional profile and diet and transcriptomic and nutrition) identified 1394 hits from November 1945 to April 2013. The search was refined by removing documents related to cancer, viruses, and so forth, and the resulting data base was purged by eliminating duplicate documents. The 646 papers obtained were critically reviewed to verify that high-throughput analyses were carried out and that a nutritional condition and hepatic expression were studied. Documents that failed to meet any of these criteria were discarded. Thus, this review covers the works related to the effects of dietary components and hepatic transcriptome in 172 papers.

## 2. DNA Microarrays

Microarray technology is a widely used approach for semiquantitative, genome-wide gene expression screening because of its easy, high-throughput data generation and affordable cost. However, it also poses some limitations that should be taking into consideration when performing this kind of analyses. 

Many of its technical aspects, such as RNA purification methods, different employed enzymes, fluorescent labels, or arrayers, are continuously being improved to enhance signal values without affecting the variability of the system [5–7]. Another important consideration comes from the fact that not all transcripts have the same level of expression and this fact may compromise response in the far extreme ranges (saturation and the lack of sensitivity in the high- and low-abundant transcripts, resp.), therefore making normalization an important issue [8]. Different strategies such as subtraction of an estimated background signal, subtracting the reference signal, smoothing (to account for nonlinear measurement effects), and others may be used. Furthermore, the probability that a false identification may occur is a real problem, when the number of tested genes gets large, as it is the case. For these reasons, software and analysis implementations are equally evolving to a fast pace [9–17]. 

In addition, the comparison of microarray platforms is questionable considering the different designs offered by manufacturers [18]. According to Genome Omnibus Organization (http://www.ncbi.nlm.nih.gov/geo/), three main companies have provided the chips most widely reported: Affymetrix, Roche/NimbleGen, and Agilent. Affymetrix GeneChip arrays use a set of 11–20 nucleotides in pairs of probes for a region of a transcript: one with perfect match and the other with a mismatch (http://www.affymetrix.com/estore/index.jsp). Roche/NimbleGen is no longer in the market. Agilent arrays use a set of 60 nucleotides (http://www.genomics.agilent.com/literature.jsp?contentType=Brochure). The selection of the capturing fragment may pose a challenge in genes with different splicing or confusion in multigene families. Therefore, comparison may be only indicative at the present time since it required standardized platforms, internal and/or external controls, and similar normalizations [19, 20]. Despite this limitation, a good agreement across different platforms has been observed and lists of platform-independent tissue-specific genes have been obtained [21].

Furthermore, the nature and extent of transcript variation differs across tissues in one individual or among individuals in part due to circadian rhythms, growth hormone signaling, immune response, androgen regulation, lipid metabolism, social stress, extracellular matrix, or epigenetic programming. In particular, this variation observed between genetically identical mice can influence the experimental design and the interpretation of data [22] particularly in studies addressing immune response, stress, amine metabolism, cell growth, ubiquitination, or hormonally regulated genes in liver [23, 24]. For these reasons, and despite many concerns raised, mRNA samples are often pooled in microarray experiments to reduce the cost and complexity of analysis of transcript profiling. Pooling RNA samples from different subjects onto a single microarray chip was found statistically valid and efficient for microarray experiments. Furthermore, optimal pooling design(s) can be found to meet statistical requirements while minimizing total cost. Appropriate RNA pooling can provide equivalent power and improve efficiency and cost-effectiveness for microarray experiments with a modest increase in total number of subjects and correct for the technical difficulty in getting sufficient RNA from a single subject [25]. Pooling hepatic RNA samples reflected the expression pattern of individual samples and properly constructed pools provided nearly identical measures of transcription response to individual RNA simple [26]. 

 Equally challenging is the gene annotation for gene clustering, genotype/phenotype correlation studies, or tissue classification when only 10% of genes have a known function and several approaches have been developed [27]. Understanding all these limitations will enable researchers to evaluate microarray results in a more cautious and appropriate manner [28]. Due to these caveats, validation of observed differences is a common practice. Real-time quantitative polymerase chain reaction (qPCR) has become the preferred option due to its high sensitivity, accuracy, high-throughput format, and relatively low costs [29]. Nonetheless, it is endorsed with its own limitations as well, namely, nonspecific amplification of non-target genes, amplification of fragments of gene families, need of reference genes, and so forth, [30, 31]. The last but not the least, such deluge of data requires integration in friendly and efficient ways to facilitate a simply use in functional genomics, not surprising that this aspect is equally evolving [32, 33]. 

## 3. Influence of Fasting/Feeding

With the end of fasting being the first step in nutrition (Table 1), we will address this issue firstly and, as expected, liver and intestine were involved. In this way, the expression profiles suggested increased cholesterol trafficking in the liver and decreased trafficking in the small intestine of fasting FVB mice. Surprisingly, in prolonged fasting, the bile salt and lipid output rates increased, with increased hepatic and intestinal lipid turnover and enhanced transintestinal cholesterol excretion. In contrast, faecal sterol loss declined sharply [34]. Other genes involved in aminoacid, lipid, carbohydrate, and energy metabolism showed significant responses to fasting in mouse liver, a response that peaked at 24 hours and was largely abated by 72 hours. The strong induction of the urea cycle, in combination with increased expression of enzymes of the tricarboxylic-acid cycle and oxidative phosphorylation, indicated a strong stimulation of amino-acid oxidation peaking at 24 hours. At this time point, fatty-acid oxidation and ketone-body formation were also induced. The induction of genes involved in the unfolded-protein response underscored the cell stress due to enhanced energy metabolism. The continuous high expression of enzymes of the urea cycle, malate-aspartate shuttle, and the gluconeogenic enzyme *Pepck* and the reappearance of glycogen in the pericentral hepatocytes indicate that amino-acid oxidation yields to glucose and glycogen synthesis during prolonged fasting. Thus, the changes in liver gene expression during fasting indicate that, in the mouse, energy production predominates during early fasting and that glucose production and glycogen synthesis become predominant during prolonged fasting [35]. 

The transition between fasting and refeeding is one of the most active transcriptional scenarios and 6000 genes were differentially expressed in mouse liver [36]. Thioredoxin binding protein-2 (TBP-2) deficient is a key regulator of peroxisome proliferator activated receptor (PPAR) alpha, and its coordinated regulation of PPAR*α* and insulin secretion is crucial in the feeding-fasting nutritional transition. Indeed, fasting-induced reduction in the expression of lipogenic genes targeted by insulin via SREBP1, such as *Fasn* and *Thrsp*, was abolished in *Tbp-2*-deficient mice, and the expression of lipoprotein lipase was downregulated, which was consistent with the lipoprotein profile. In fed *Tbp-2-*deficient mice, there were elevated expressions of PPAR*α* and PPAR*γ* coactivator-1*α* proteins and their target genes *Cd36*, *Fabp2*, *Acot1*, and *Fgf21*, whereas the fasting-induced upregulation of PPAR*α* was attenuated [37]. Thus, fasting is inducing a complex transcriptional response where PPAR*α* and TBP-2 are important agents.

Rats are also a species in which these studies have been published. Gene expression profiles of energy metabolism-related genes in the livers of rats allowed to feed for 6 h after 18 h fasting, and those in 24 h fasting rats were different. In addition, refeeding induced upregulation of the genes encoding immunoproteasome components [38]. In Clock mutant mice bred under constant light to attenuate the endogenous circadian rhythm, hepatic *Per1*, *Per2*, and* Dec1* expressions were significantly increased while that of *Rev-erb*α** decreased within 1 h of feeding after 24 h fasting. An intraperitoneal injection of glucose combined with amino acids reproduced a similar hepatic response [39]. Following feeding, 42 proteins involved in protein synthesis increased their abundance in polysomes, and this may contribute to explain the increases in protein and RNA content in this situation [40].

## 4. Influence of Caloric Restriction

In rats, a caloric restriction (CR) of 30% of calories showed altered hepatic expression of genes involved in the stress response, xenobiotic metabolism, and lipid metabolism. Gene expressions involved in stress response and xenobiotic metabolism were regulated in a growth hormone/insulin growth factor-1-dependent manner, while those involved in lipid metabolism were independent. Moreover, CR enhanced the gene expression involved in fatty acid synthesis after feeding and those encoding mitochondrial beta-oxidation enzymes during food shortage, probably via transcriptional regulation by peroxisome proliferator-activated receptor alpha. Thus, caloric restriction promotes lipid utilization through hepatic transcriptional alteration and may prevent hepatic steatosis [41]. In this sense, upregulation of the three *Nr4a* receptors was observed in Brown Norway liver rats and based on the proposed roles of the NR4A nuclear receptors in sensing and responding to changes in the nutritional environment, and in regulating glucose and lipid metabolism and insulin sensitivity, it was hypothesized that these proteins may participate in caloric restriction adaptation [42]. The use of different levels of restriction (5%–30% of calories) had an important impact on gene expression, particularly lipid metabolism. The fact that these transcriptional changes were even observed with the lowest level that did not modify body weight emphasizes the sensitivity of transcriptomics studies [43].

The length of dietary restriction has been shown to play a role. Both just 4 weeks and long-term-caloric restriction reversed the majority of aging-induced changes in hepatic gene expression, mainly increased inflammation, cellular stress, fibrosis, and reduced capacity for apoptosis, xenobiotic metabolism, normal cell cycling, or DNA replication [44]. This indicates that the benefits of caloric restriction are established rapidly. However, the influence of experimental design may influence results. In fact, two acute progressive feed restriction regimens causing identical diminution of body weight (19%) but differing in duration (4 versus 10 days) led to distinct patterns of differentially expressed genes in liver. Albeit some major pathways of energy metabolism were similarly affected (particularly fatty acid and amino acid catabolism), the longer regimen also induced deregulation of circadian rhythms [45]. 

The hepatic transcript profile in wild-type mice undergoing caloric restriction presents some overlaps with those of agonists of lipid-activated nuclear receptors, including PPAR*α*, liver X receptor, and their obligate heterodimer partner, retinoid X receptor. In fact, 19% of all gene expression changes were dependent on PPAR*α*, including *Cyp4a10* and *Cyp4a14*, involved in fatty acid omega-oxidation, acute phase response genes, and epidermal growth factor receptor but not on PGC-1*α*. Based on these observations, it is hypothesized that some effects of caloric restriction are mediated by PPAR*α* [46] in agreement with the data observed in rats. DNA microarray analysis was used to identify genes upregulated in the liver of caloric restricted mice and Ames dwarf mice, which are deficient in growth hormone, prolactin, and thyroid-stimulating hormone and live significantly longer than their normal siblings. A search for sequence similarity among those genes revealed the presence of consensus sequence motifs named dwarfism and caloric restriction-responsive elements. This has been exploited to prepare a highly sensitive bioassay to identify agents mimicking the antiaging effects of caloric restriction [47]. Beef cattle genetically selected for feed efficiency showed 161 hepatic genes that were differentially expressed. These genes were involved in seven gene networks affecting cellular growth and proliferation, cellular assembly and organization, cell signaling, drug metabolism, protein synthesis, lipid metabolism, and carbohydrate metabolism [48]. Overall, food restriction and efficiency are complex processes that are highly regulated.

The influence of major nutrient components-, carbohydrates, fat, and proteins- is summarized in Table 2. 

## 5. Effects of Carbohydrate Content of Diets

Diets of high glucose content modified expressions of genes related to thiol redox, peroxisomal fatty acid oxidation, and cytochrome P450 in C57BL/6J mice, contributing to enhance oxidative stress [49]. A high sucrose diet in Nagoya-Shibata-Yasuda, but not in C3H mice, increased hepatic expression levels of *Pparg2*, as well as *G0s2*, a target of *Pparg*, which are known to be adipocyte-specific genes. In contrast, hepatic levels of *Kat2b* (transcriptional regulation), *Hsd3b5* (steroid hormone metabolism), and *Cyp7b1* (bile acid metabolism), initially lower in Nagoya mice, were further decreased in this mouse strain receiving high sucrose diet [50]. Dietary sweet corn feeding in mice had a profound influence on 1600 gene expression levels, some of them related to cell proliferation and programmed cell death. In the Wnt signaling pathway, which is involved in cell proliferation, the levels of Jun and beta-catenin expression were downregulated, while those of *Rb* and *p53*, negative regulators of the cell cycle, or those of *Bok, Bid*, and *Casp4* involved in apoptosis were increased [51]. These results point out to heterogeneity in response to these diets. 

 Rats fed a 20% maple syrup diet for 11 days showed significantly lower values of the hepatic function markers than those fed a 20% sugar mix syrup diet. A DNA microarray analysis revealed that the expression of genes for the enzymes of ammonia formation was downregulated in the liver of the maple syrup diet [52]. Short-chain fructooligosaccharide changed the expression of PPAR*α*, phytanoyl-CoA 2-hydroxylase 2, lipoprotein lipase, and tyrosine aminotransferase, farnesoid X receptor (FXR) target genes in the rat liver [53]. The activation of lipoprotein lipase and FXR-target genes may participate in the lipid lowering effects of those compounds [54]. Other sources of carbohydrates such as hypoallergenic wheat flour when fed to rats upregulated genes known to respond to the interferon-gamma signal which may be related to possible oral immunotolerance resulting after feeding this flour [55]. Therefore, carbohydrates elicit a wide range of responses.

## 6. Effect of Dietary Fat Content

This is an aspect that has been widely analyzed in terms of amount and type of fat. Surprisingly, the influence of a fat-free diet has been scarcely studied. One study in rat liver consuming this type of diets showed that emerin, an integral protein of the inner nuclear membrane, was highly expressed independently of the sterol regulatory element binding regulation pathway [78].

### 6.1. Studies on High Fat Diet

These are aspects profusely tackled by different authors with different approaches in terms of length of intervention and amount of fat. In a postprandial regimen, a bolus of 5 mL of extravirgin olive oil given to male rats induced significant changes of hepatic *A2m*, *Slc13a5*, and *Nrep* mRNA expressions which were significantly associated with postprandial plasma triglycerides [56].

One-week administration of a high-fat diet reduced hepatic *Cyp3a* expression in obese mice. However, changes in nuclear receptors involved in the transcriptional regulation of this gene were not correlated with its expression. Obese mice induced by gold thioglucose administration exhibited a different expression profile of hepatic P450s with no significant change in *Cyp3a*. High-fat diet-induced changes in energy metabolism, which eventually result in obesity, modulate the hepatic expression profile of P450s, particularly *Cyp3a* [57].

Long-term administration of high fat diets (HFD) has been widely explored to develop nonalcoholic fatty liver disease (NAFLD) or insulin resistance [79] and the linking association between both diseases [80]. The former was induced in 129S6 male mice fed diets containing 40% fat for 15 weeks and associated with increased hepatic transcription of genes involved in fatty acid uptake, intracellular transport, modification, and elongation, whilst genes involved in beta-oxidation and lipoprotein secretion were, paradoxically, also upregulated. NAFLD developed despite a downregulation of transcription of the gene encoding stearoyl-coenzyme A desaturase 1 (*Scd1*) and uncoordinated regulation of transcription of *Scd1* and the gene encoding sterol regulatory element binding protein 1c (*Srebp1c*) transcription [58]. *Scd1* was associated with the expression of patatin-like phospholipase domain containing 3 (PNPLA3). The latter also associated other lipogenic genes (*Me*1 and *Spot*14) and increased its expression in Western-type diet-fed C57BL/6 mice and LDLR−/− mice [81]. Overexpression of the genes related to lipid metabolism, adipocyte differentiation, defense, and stress responses was also noticeable in the NAFLD of obese rodents. In this way, livers are supplied with large amounts of free fatty acids either through increased fatty acid biosynthesis or through decreased fatty acid oxidation, which may lead to increased mitochondrial respiratory activity [26, 82]. These findings may not be universal for all kind of dietary fats since C57BL/6 mice fed a high-fat lard diet showed that genes encoding proteins involved in lipogenesis and xenobiotic metabolism (glutathione S-transferases mu1 and pi1 and selenium-binding protein 2) were downregulated, whereas genes involved in fatty acid oxidation were upregulated [83]. Furthermore, a mild high-fat (15%) diet caused differential regulation of 200 genes, while a severe one caused the expression of 788 genes in C57BL/6 and 1010 genes in APOE3Leiden (E3L) mice. Lipid metabolism and inflammation, the latter as determined by “immune/defense response and detoxification” processes, were strongly affected by genotype and diet. The severe high-fat (15%, w/w), high cholesterol (10%), and cholate (0.5%, w/w) diet reduced expression of genes involved in bile acid, sterol, steroid, fatty acid, and detoxification metabolism. This common regulation of genes underlying lipid and detoxification processes suggests a defense mechanism to protect against the accumulation of toxic endogenous lipids and bile acids [84]. These results indicate that the fat source and amount may play an important role on the outcome and it emphasizes the flexibility of genome to cope with different circumstances.

As mentioned above, high fat diets stimulated inflammatory mechanisms [58, 83]. The latter aspect has been related to the fact that this diet promoted infiltration of hepatic tissue by leukocytes, leading to elevated expression of immune-associated transcripts, particularly of genes encoding components of the toll-like receptor signaling pathway (e.g., *Irf5* and *Myd88*). In some strains (e.g., NZB/BINJ, B6), 50%–60% of transcripts elevated by high fat diet might be due to hepatic infiltration by these cell types. Interestingly, DBA mice appeared to exhibit resistance to localized hepatic inflammation associated with atherogenic diet. This emphasizes that the effect is genetically controlled and sensitive to both strain and sex [59]. A more exacerbated transcriptional response was observed in high-fat diet-fed apolipoprotein E2 mice, and genes encoding chromatin-remodeling enzymes, such as jumonji C-domain-containing histone demethylases that regulate histone H3K9 and H3K4 trimethylation (*H3K9me3*, *H3K4me3*), were significantly altered in steatotic livers. The global methylation status in lipid-accumulated mouse primary hepatocytes by ChIP-on-chip analysis showed that hepatic lipid accumulation induced aberrant *H3K9me3* and *H3K4me3* status in peroxisome proliferator-activated receptor alpha and hepatic lipid catabolism network genes, reducing their mRNA expression compared with nontreated control hepatocytes [85]. Aging is also an important variable since hepatic gene expression changes were more pronounced in the context of aged C57BL/6J mice [86], and the molecular mechanisms underlying high-fat feeding or aging which mediated insulin resistance were not identical. 

As already mentioned, responses to diets frequently differ among different strains of mice. In this sense, the C57BL/6J-fed mice exhibited signs of insulin resistance, while the A/J mice did not after a long-term consumption of a high fat diet [87]. These findings were considered related to differential gene expressions [88], and therefore these experiments pursued. As a consequence, resistance to steatosis in A/J mice fed a high fat diet was associated with a coordinated upregulation of 10 genes controlling peroxisome biogenesis and beta-oxidation and an increased expression of the elongase *Elovl5* and desaturases *Fads1* and *Fads2*. In agreement with these observations, peroxisomal beta-oxidation was increased in livers of A/J mice, and lipidomic analysis showed increased concentrations of long chain fatty acid-containing triglycerides, arachidonic acid-containing lysophosphatidylcholine, and 2-arachidonoylglycerol, a cannabinoid receptor agonist. The anti-inflammatory CB2 receptor was the main hepatic cannabinoid receptor and was highly expressed in Kupffer cells. A/J mice had a lower proinflammatory state as determined by lower plasma levels and IL-1*β* and granulocyte-CSF and reduced hepatic expression of their mRNAs, which were found only in Kupffer cells. This suggests that increased 2-arachidonoylglycerol production may limit Kupffer cell activity [89]. Moreover, high-fat diet feeding in the A/J, but not in the C57Bl/6, mouse livers upregulated 13 oxidative phosphorylation genes without changes in ATP production, which indicated increased uncoupling of the A/J mitochondria [90]. Thus, variations in the expression of peroxisomal beta-oxidation and of anti-inflammatory lipid- and oxidative phosphorylation activity-involved genes may protect A/J mouse livers against the initial damages induced by high-fat diet that may lead to hepatosteatosis. In an other approach, exaggerating fat and lean mouse strain differences with chronic high fat feeding revealed a distinct gene expression profile of line, fat depot, and diet-responsive inflammatory, angiogenic, and metabolic pathways [91]. In an other study, different phenotypes of genetically homogenous C57Bl/6 mice fed a high fat diet for 9 months were observed. While most become obese and diabetic, a significant fraction remains lean and diabetic or lean and nondiabetic. Obesity/diabetes was associated with preserved hepatic lipogenic gene expression and increased plasma levels of very low density lipoprotein. In contrast, the lean mice showed a strong reduction in the expression of hepatic lipogenic genes, in particular of *Scd1*, a gene linked to sensitivity to diet-induced obesity; the lean and nondiabetic mice presented an additional increased expression of eNos in liver. There was a progressive establishment of the different phenotypes, and development of the obese phenotype involved reexpression of *Scd1* and other lipogenic genes [92].

The characterization of chronic response to HFD has been addressed in rats as well. Among 130 genes found, sterol regulatory element binding factor 1 and stearoyl-coenzyme A desaturase 1 had upregulation, whereas others like peroxisome proliferator-activated receptor, carnitine palmitoyltransferase 1, and 3-hydroxy-3-methylglutaryl-coenzyme A reductase had repressed expression. Metabolomic analysis showed that tetradecanoic acid, hexadecanoic acid, and oleic acid had elevation while arachidonic acid and eicosapentaenoic acid had decreased content in HFD rat livers. Glycine, alanine, aspartic acid, glutamic acid, and proline contents were decreased. In obesity-prone and obesity-resistant rats receiving high fat diet, fatty acid metabolism, Krebs cycle, and amino acid metabolism were also the origin of metabolites differing between the two phenotypes [93]. These integrative results revealed that, in this model, fatty acid utilization through beta-oxidation was inhibited and lipogenesis was enhanced by this type of diets [60].

Besides the differences among genotypes, epigenetics may play a role in response to HFD. In this regard, these studies have been addressed in C57BL6/J mice using a microchip to test imprinted genes [94] or in Japanese macaques. In the latter case, consumption of a maternal high-fat (35% fat) diet resulted in increased fetal liver triglycerides and histologic correlates of nonalcoholic fatty liver disease, accompanied by a hyperacetylation at K14 of Histone 3 and depletion of histone deacetylase 1. Using ChIP differential display PCR to link fetal modifications of Histone 3 acetylation with alterations in gene-specific expression, it was found a 40% increase in the expression of several genes including glutamic pyruvate transaminase (alanine aminotransferase) 2 (*Gpt2*), *Dnaja2*, and *Rdh12*, while *Npas2*, a peripheral circadian regulator, was downregulated [95]. 

Moreover, the combination of social stress and western diet resulted in significant perturbations in lipid regulation, including two key features of the metabolic syndrome: increased plasma levels of non-HDL cholesterol and intrahepatic accumulation of triglycerides. These effects were accompanied by a number of changes in the expression of hepatic genes involved in lipid regulation and transcriptional activity of LXR, SREBP1c, and ChREBP [96].

### 6.2. Influence of Nature of Fat

Recognized as an important factor, a growing number of studies are addressing this issue to establish the influences of monounsaturated, polyunsaturated fatty acid-containing diets. In this way, substitution of dietary monounsaturated or polyunsaturated fatty acid (olive oil and menhaden oil) for carbohydrate reduced hepatic expression of SREBP-1c, with concomitant reductions in hepatic triglyceride content, lipogenesis, and expression of enzymes related to lipid synthesis in corpulent James C. Russell (JCR:LA-cp) rats. Unexpectedly, this substitution increased expression of many peroxisomal proliferator-activated receptor-dependent enzymes mediating fatty acid oxidation [61]. Olive substitution for butter (10% of total energy) for two weeks modulated several genes related to lipolysis or lipogenesis in normal rats [62] and newly identified genes from other metabolisms (*Fsp27* and *Syt1*) in apoE-deficient mice [65]. The menhaden oil diet further increased expression of these enzymes. Induction of SREBP-1c by insulin was dependent on LXR*α*. Expression of mRNA encoding fatty acid translocase and ATP-binding cassette subfamily DALD member 3 was also increased in livers of corpulent JCR rats, indicating a potential role for these fatty acid transporters in the pathogenesis of disordered lipid metabolism in obesity [61].

Different transcriptomic response was observed among different long chain polyunsaturated fatty acids. The n-3 polyunsaturated fatty acid containing diets, provided as fish oil, regulated lipolytic and lipogenic gene expression and the tissue specificity of this regulation in mice [63], rats [62], and in JCR:LA-cp rats [61]. These diets also increased bile and cholesterol excretion controlling cholesterol metabolism by inducing cholesterol 7alpha-hydroxylase and its upstream transcription factors: D-site binding protein and liver X receptor-alpha in mice [97], and independently of LXR transcription in JCR:LA-cp rats [61]. These fatty acids regulated the expression of genes involved in many other pathways such as oxidative stress response and antioxidant capacity, cell proliferation, cell growth and apoptosis, cell signaling, and cell transduction. They act as cellular regulators [63].

A balance of dietary n6 and n3 PUFA could have profoundly different effects on metabolism and cell signaling as indicated by the consumption of n6 (rich in 20:4n6), n3 (rich in 20:5n3, 22:5n3, and 22:6n3), and a combination of the two. The combination had unique effects on murine hepatic transcripts involved in cytoskeletal and carbohydrate metabolism, whereas n6 affected amino acid metabolism via CTNB1 signaling. All three diets affected transcripts linked to apoptosis and cell proliferation, with the evidence that n3 may have increased apoptosis and decreased cell proliferation via various transcription factors, kinases, and phosphatases. The three diets affected lipid transport, lipoprotein metabolism, and bile acid metabolism through diverse pathways. n3 activated cytochromes P450 that form hydroxylated fatty acids known to affect vascular tone and ion channel activity. Fatty acid synthesis and delta 9 desaturation were downregulated by the combination, implying that a mixture of 20:4n6, 20:5n3, and 22:6n3 is most effective at downregulating synthesis, via INS1, SREBP, PPAR*α*, and TNF signaling. Heme synthesis and the utilization of heme for hemoglobin production were likely affected by n6 and n3. Relative to other groups, n3 increased numerous transcripts linked to combating oxidative stress such as peroxidases, an aldehyde dehydrogenase, and heat shock proteins, consistent with the major LC-PUFA in n3 (20:5n3, 22:5n3, 22:6n3) being more oxidizable than the major fatty acids in n6 (20:4n6) [64].

More sophisticated seems to be the different effect displayed by the isomers of linoleic acid. Female mice consuming 0.5% of t10, c12-conjugated linoleic acid (CLA) isomer showed reduced expression of fatty acid oxidation genes including flavin monooxygenase (FMO)-3, cytochrome P450, carnitine palmitoyl transferase 1a, acetyl CoA oxidase, and PPAR*α* and increased expression of fatty acid synthase. Thus, both decreased fatty acid oxidation and increased fatty acid synthesis seem to contribute to the CLA-induced fatty liver [98]. Although other mechanisms may be involved, this isomer showed significant associations among ten-gene (*Fsp27*, *Aqp4*, *Cd36*, *Ly6d*, *Scd1*, *Hsd3b5*, *Syt1*, *Cyp7b1*, and* Tff3*) expressions and the degree of hepatic steatosis in ApoE-deficient mice fed a Western-type diet [65]. These effects were not observed in Syrian Golden hamsters receiving this isomer [66]. Likewise, different responses to the c9, t11-CLA isomer were observed between ApoE- and leptin-deficient mice on insulin signaling and lipogenic pathways, which were adversely affected in *Apoe*-deficient mice but improved insulin sensitivity in leptin-deficient mice [67]. These results again emphasize the relevance of the genetic background on the outcome.

The response to these diets may be conditioned by feeding regimen. Indeed, it can be observed that hepatic *Elovl3* mRNA expression follows a distinct diurnal rhythm in mature male mice and that the animals that were exclusively fed during the day for 9 days displayed an inverted expression profile. Thus, *Elovl3* expression in mouse liver is under strict diurnal control by circulating steroid hormones such as glucocorticoids and androgens. *Elovl3* expression was increased and decreased in peroxisomal transporter ATP-binding cassette, subfamily D(ALD), member 2 knockout, and transgenic mice, respectively, which indicates a tight regulation between very long chain fatty acid synthesis and peroxisomal fatty acid oxidation [99].

High fat diets have been the common ground to test the influence of other compounds. In male Sprague-Dawley rats, plasma triacylglycerol and phospholipid concentrations were significantly lower in the groups consuming 21% high-fat diet as lard supplemented with 0.05% oleanolic acid (50 mg/kg/day) or 0.45% pomace ethanolic extract (450 mg/kg/day). Reduced expression levels of lipogenic genes including acetyl-CoA carboxylase and glycerol-3-phosphate acyltransferase of gluconeogenesis and inflammatory cytokines were observed in the oleanolic and pomace extract groups. In this way, these compounds could ameliorate obesity-induced insulin resistance [100]. Likewise, rats fed a high-fat diet and mulberry (*Morus alba*) leaves showed reduction in plasma lipids, which could be explained by the upregulated expressions of genes involved in the response to oxidative stress and in alpha-, beta-, and omega-oxidation of fatty acids, related to the peroxisome proliferator-activated receptor signaling pathway and downregulation of the genes involved in lipogenesis [101]. 

Many compounds have been shown to antagonize the fatty degenerative state of the hepatocytes induced by high fat diets in mice. In this regard, licorice flavonoid oil upregulated the expression of genes coding for beta-oxidation and downregulated those coding for fatty acid synthesis [102]. Likewise, 0.1% lipoic acid increased beta-oxidation and decreased cholesterol synthesis and oxidative stress by increasing those of free radical scavenger enzyme gene expression in liver of C57BL/6 mice [103]. In rats fed a high-fat/high-fructose diet, compounds present in *Eucommia ulmoides* tea (4 or 20 g/L extract) also increased expression of genes involved in hepatic alpha-, beta-, and omega-oxidation, mainly related to the peroxisome proliferator-activated receptor alpha and delta signaling pathways [104]. Furthermore, combined quercetin and resveratrol supplementation resulted in significant restoration of gene sets in pathways of glucose/lipid metabolism, liver function, cardiovascular system, and inflammation/immunity, which were altered by high fat diet feeding [105]. Oleuropein at 0.03% also reduced the hepatic expression of the genes codifying for hepatic fatty acid uptake and transport, those involved in the oxidative stress responses and detoxification of lipid peroxidation products and proinflammatory cytokine genes in C57BL/6N mice. Several transcription factors that bound to the promoters of the genes regulated at least threefold by oleuropein were implicated in the lipogenesis, inflammation, insulin resistance, fibrosis, and cell proliferation and differentiation, which implies that the mechanisms that underlie the beneficial effects of oleuropein on fatty liver may be multifactorial [106]. This complexity has been proposed after 5% psyllium administration where the expression levels of genes involved in fatty acid oxidation and lipid transport were significantly upregulated in the skeletal muscle, and this effect could create a slightly insufficient glucose state in the liver [107].

### 6.3. Influence of Dietary Cholesterol Content

Providing dietary cholesterol to mice has been a strategy to induce nonalcoholic fatty liver disease. The percentage of cholesterol varied in a wide range depending on studies. Inoue et al. used very high percentages ranging from 10% to 80% [68] and observed a clear fatty liver phenotype after a 12-week intervention. In these conditions, protein expression of cAMP response element-binding protein (CREB), an upstream molecule of PPAR*γ*, was suppressed, while gene expressions involved in lipid metabolism, adipogenesis-related genes, PPAR*γ*, and its targeted gene, *Cd36*, were upregulated in the liver.

Using rather modest dietary cholesterol contents (0.0% versus 0.5% cholesterol wt/wt), 69 unique hepatic UniGene clusters were modified (37 downregulated and 32 upregulated). When six downregulated genes were analyzed in transgenic mice overexpressing truncated nuclear and active forms of SREBP-1a and SREBP-2, all were induced. In mice treated with the LXR agonist TO901317, 13 of the 32 cholesterol-upregulated genes were also LXR-activated. In this way, six novel dietary cholesterol-regulated genes were identified, three putative SREBP target genes (calcium/calmodulin-dependent protein kinase 1D, fatty acid binding protein 5, and proprotein convertase subtilisin/kexin 9) and three putative LXR target genes (a disintegrin and metalloprotease domain 11, apoptosis-inhibitory 6, and F-box-only protein 3) [69].

Dietary cholesterol also induced hepatic genes involved in drug metabolism and acute inflammation (including three genes of the serum amyloid A family, three major histocompatibility class II antigen genes, and various cytokine-related genes) in C57BL/6J mice. It downregulated cholesterol biosynthesis as expected [70, 71]. In apoE-KO mice, a 1.25% cholesterol diet also regulated hepatic triglyceride metabolism through a suppression of *Lipin1* and *Lipin2* and by decreasing peroxisome proliferator-activated receptor-gamma coactivator 1*α*, which upregulates the transcription of *Lipin1 *[72]. Therefore, cholesterol may induce an inflammatory response and modulate hepatic triglyceride.

A genetic locus (Diet1) was responsible for the resistance to diet-induced hypercholesterolemia and atherosclerosis phenotype in B6By compared to B6J mice. Comparing hepatic expression profiles from both strains, B6By mice showed elevated levels for key bile acid synthesis proteins, including cholesterol 7alpha-hydroxylase and sterol-27-hydroxylase, and the oxysterol nuclear receptor liver X receptor alpha. Other genes involved in bile acid metabolism (farnesoid X receptor, oxysterol 7alpha-hydroxylase, sterol-12alpha-hydroxylase, and hepatic bile acid transporters) were also modified. Thus, B6By strain owns a higher rate of bile acid synthesis and transport [108]. A different response to dietary cholesterol among rats allowed identifying a sensitive locus (Dihc2) to chromosome 14. Dihc2 was linked to a region including 33 genes and predicted transcripts and identified RGD1309450 predicted, a homologous gene of SMEK2, as a strong candidate for responsiveness to dietary cholesterol [109].

As shown for high fat diets, cholesterol-induced changes have been used to test the influence of other substances. In this regard, *Lactobacillus brevis* 119-2 given to Sprague-Dawley rats kept on a cholesterol diet lowered serum cholesterol and ameliorated fatty liver due to inhibition of 3-hydroxy-3-methylglutaryl-CoA reductase activity by insulin induced gene protein, induction of catabolism of cholesterol to bile acid by *Cyp7a1,* and overexpression of the LDL receptor gene. In contrast, *Lactobacillus acidophilus* ATCC 43121 increased high density lipoprotein cholesterol levels by inducing overexpression of the ATP-binding cassette sub-family A member 1 (*Abca1*) and Angiopoietin-like 3 (*Angptl3*) genes [110].

## 7. Influence of Dietary Bile Acid Contents

Cholate administration to mice induced expression of genes involved in extracellular matrix deposition in hepatic fibrosis, including five collagen family members, collagen-interacting proteins, and connective tissue growth factor. Thus, this dietary supply may induce fibrogenesis [70].

Chenodeoxycholic acid is considered the natural farnesoid X receptor (FXR) agonist, and when added to HepG2 cells, it increased the expression of very low density lipoprotein receptor. In FXR-deficient mice, no such response was observed, which indicated that the action of chenodeoxycholic acid on this gene is clearly dependent on FXR [111].

## 8. Effects of Protein Content and Quality of Proteins

Compared to the influence of fat, this aspect has received less attention. The gene expression profiles among rats fed on 12% casein, 12% gluten, and protein-free diets for one week revealed that a few hundred genes in the liver were up or downregulated by more than twofold after feeding of the gluten or the protein-free diet. Among these, the induction of genes for synthesis and catabolism of cholesterol by gluten feeding were observed [112]. The effect of consumption of an enzymatically produced, hypoallergenic wheat flour on gene expression profiles in rats confirmed the safety of this novel food product [113, 114]. The source of protein either soy or casein had a profound impact on hepatic expressions of genes related to lipid metabolism, transcription factor, and antioxidization enzymes [73]. In lipid metabolism especially, the downregulated genes were related to fatty acid synthesis and the upregulated genes are related to cholesterol synthesis and steroid catabolism [74]. Similar findings of amino acid impact were observed when studying that consumption of diets containing more than 4% Leu in 6% protein content resulted in growth retardation and reduced liver weight, whereas the administration of the same dose of Leu with 12% or 40% protein did not affect Sprague-Dawley rats. Using this approach, 6 candidate gene markers were identified in liver [115].

The supply of specific amino acids such as the branched-chain ones reversed the gene expression changes observed in cirrhotic Wistar rats. Among these, it was observed the downregulation of fatty acid translocase (*Fat*/*Cd36*), glutamine synthetase, and pyruvate dehydrogenase kinase isoenzyme 4, believed to promote lower uptake of fatty acids, lower ammonia incorporation, and higher uptake of glucose, and thus to provide an energy source without using the branched amino acids. In this way, their catabolism and skeletal muscle protein would be slowed, maintaining their concentrations in blood [75].

More severe approaches of amino acid deprivation have been adopted. In C57BL/6 mice on a methionine- and choline-deficient diet for 6 weeks, it was showed increased ALT, histological features of nonalcoholic steatohepatitis (NASH), and oxidative liver damage with increases in 4-hydroxynonenal and 3-nitrotyrosine. Of the genes analyzed, the GPx family, Fmo2, and peroxiredoxins were significantly upregulated, whereas *Scd1*, Catalase, and *Serpinb1b* were significantly downregulated. Thus, oxidative stress-related genes were differentially expressed in the livers of mice with diet-induced NASH [116]. These diets downregulated the expression of *Riz1*, an activity associated with greater H3 lysine 9 methylation in RIZ1 target genes. Thus, RIZ1 is a critical target of methyl donors as represented by methionine and choline [76].

 An even more severe regimen was used in male F344 rats, fed a diet deficient in L-methionine and devoid of folic acid and choline to induce hepatocellular carcinomas, and it was shown alterations of components of the DNA methylation machinery, namely, *de novo* DNA methyltransferases (*Dnmt3a* and 3b), maintenance DNA methyltransferase (*Dnmt1*), and methyl CpG binding proteins (MBDs) by both transcriptional and posttranscriptional mechanisms during early stages of hepatocarcinogenesis [77]. 

The amount of protein was explored in pregnant sows to test the influence on their offspring. In this regard, they were fed either a gestational low protein diet (LP, 6%) or an adequate protein diet (AP, 12%) and their offspring was nursed by foster sows receiving standard diets and after weaning, they receive these diets. Differential expression of genes related to lipid metabolism (e.g., fatty acid metabolism, biosynthesis of steroids, synthesis and degradation of ketone bodies, fatty acid elongation, and bile acid synthesis) and cell cycle regulation (e.g., mitotic roles of PLK, G_1_/S checkpoint regulation, and G_2_/M DNA damage checkpoint regulation) were observed. The transcriptomic modulations point to persistent functional demand on the liver towards cell proliferation in the LP group but not in the AP group at identical nutritional conditions during postnatal life due to divergent “programming” of the genome [117].

## 9. Influence of Dietary Salt Content

Feeding mice with a high-salt diet *ad libitum* for over 2 weeks advanced the phase of clock gene expression by about 3 hours in the liver, but did not change circadian feeding, drinking, and locomotor rythms. Many genes related to metabolism in the liver were also advanced. Blood glucose uptake increased more rapidly after consuming this diet than the control diet. Moreover, phloridzin, a specific inhibitor of SGLT1 transporter, prevented the increased glucose transporter expression in the jejunum and phase advancement in the livers. Thus, the increased glucose absorption induced by dietary high salt may alter the food entrainment of peripheral molecular circadian rhythms [118].

## 10. Vitamins

Vitamins of hydro- and liposoluble categories or their analogues have been explored. Vitamins of B group are the first to be considered. In this way, administration of methylated niacin (vitamin B_3_) or trigonelline mitigated diabetes in diabetic Goto-Kakizaki rats by decreasing oxidative stress, particularly the expression of NADPH oxidase and mitochondrial electron transfer system [119]. Folate deficiency modified expression of genes involved in fatty acid metabolism, DNA synthesis, and circadian rhythm [120].

Vitamin C supplementation rescued the shorter mean life span of Wener-deficient mice and reversed several age-related abnormalities in liver such as endothelial defenestration, genomic integrity, and inflammatory status. Likewise, vitamin C normalized phosphorylation of Akt kinase-specific substrates, NF-*κ*B, protein kinase C*δ*, and Hif-1*α* transcription factor levels. It also increased PPAR*α*. Vitamin C decreased hepatic genes normally upregulated in cancer, and it increased genes involved in tissue injury response and adipocyte dedifferentiation in obese mice. This vitamin did not have such effect on wild-type mice [121]. Moreover, vitamin C (0.056 mg/g of body weight) for 1 week induced overall energy metabolism as well as radical scavenging pathways in the fasting state and more profoundly in the refeeding state [36]. Thus, vitamin C supplementation may help prevent oxidative stress and could be beneficial for patients with de Werner syndrome.

Vitamin E supplementation for 290 days downregulated scavenger receptor *Cd36*, coagulation factor IX, and 5-alpha-steroid reductase type 1 mRNA levels, while hepatic gamma glutamyl-cysteinyl synthetase was significantly upregulated in male rat livers. Measurement of the corresponding biological endpoints such as activated partial thromboplastin time, plasma dihydrotestosterone, and hepatic glutathione supported the gene chip data [122]. When dietary alpha-tocopherol was studied, the expression of 389 hepatic genes by a factor of 2 or higher was found modified. Of these, 121 genes were involved in transport processes and twenty-one thereof in vesicular trafficking. Upregulation of syntaxin 1C, vesicle-associated membrane protein 1, N-ethylmaleimide-sensitive factor, and syntaxin binding protein 1 suggests a role of alpha-tocopherol in the vesicular transport not only influencing its own absorption and transport but also explaining other dysfunctions observed in severe alpha-tocopherol deficiency [123].

Vitamin K1, orally administrated, suppressed hepatic expression of genes involved in the acute inflammation in the rat either induced by vitamin K-deficient diets or by lipopolysaccharide administration [124].

## 11. Effects of Different Oligoelements

Zinc-deficiency in rats had an important influence on hepatic gene expression particularly those related to lipid metabolism. Indeed, lipolysis and mitochondrial as well as peroxisomal fatty acid degradation were downregulated, whereas those needed for *de novo* fatty acid synthesis and triglyceride assembly were increased [125]. Likewise, iron deficiency in this animal had profound impact on hepatic transcription by upregulating genes involved in cholesterol metabolism, reticulum-specific apoptosis, amino acid, and glucose metabolism, while genes related to lipid metabolism were significantly downregulated [126].

Mice receiving dietary supplementation of 2.1 mg/kg selenium-enriched broccoli showed increased *IkBalpha*κ*B*, *Hsp86,* and *Gadd45* gene transcripts in liver. In addition, analysis of the binding of liver nuclear proteins demonstrated enhanced binding of transcription factors p53, NF-*κ*B, and AP-1 to their cis-acting elements. In this way, selenium-enriched broccoli activates certain proapoptotic genes linked to p53, NF-*κ*B, and stress signal pathways [127].

## 12. Minor Dietary Components of Foods

### 12.1. Minor Components of Extra Virgin Olive Oil

Extra virgin olive oil is a complex oleous mixture obtained from olive fruits that contains more than three hundred minor components generally named unsaponifiable fraction [128]. To explore its role in apoE-deficient mice, different oils were prepared. In the absence of hepatic steatosis and inflammation, unsaponifiable fraction-enriched olive oil upregulated orosomucoid, serum amyloid A2, *Fabp5,* and *Mt2* and downregulated several proteases [129]. When especifically maslinic acid-enriched oils were tested, *Cyp2b9*, *Cyp2b13*, and *Dbp* expressions appeared significantly increased, and Marco was significantly decreased in apoE-deficient mice. Dbp was upregulated to an extent depending on the genetic background of the mice and negatively associated with the expression of *Marco*, a gene strongly upregulated by the absence of apoE [130]. Secoiridoids are also minor components of extra virgin olive oil belonging to aqueous soluble category. Secoiridoids such as oleuropein aglycon and decarboxymethyl oleuropein aglycon activated endoplasmic reticulum stress, the unfolded protein response, spermidine and polyamine metabolism, sirtuin-1 and NRF2 signaling, and AMPK and suppressed genes involved in the Warburg effect and the self-renewal capacity of cancer stem cells. Therefore, they showed antiaging transcriptomic signatures [131]. Overall, the use of transcriptomic analyses has widened the understanding of biological effects of the unsaponifiable fraction present in olive oil. Thus, the term “monounsaturated fatty acid-enriched oil” encompassing oils such as olive, high-oleic sunflower, peanut, and almond, based on the high percentage of oleic acid no longer appears appropriate for describing their biological properties since they have different unsaponifiable composition and this fraction is highly active.

### 12.2. Coffee

In unfiltered coffee brews, cafestol is a present diterpene and having the most potent cholesterol-elevating action known in the human diet. Cafestol-treated APOE3Leiden mice showed alterations in hepatic expression of genes involved in lipid metabolism and detoxification, many of which were regulated by the nuclear hormone receptors farnesoid X receptor (FXR) and pregnane X receptor (PXR). Cafestol, as an agonist ligand for FXR and PXR, downregulates expression of the bile acid homeostatic genes *Cyp7a1*, sterol 12alpha-hydroxylase, and Na^+^-taurocholate cotransporting polypeptide in the liver of wild-type but not in FXR-deficient mice, thereby suggesting a role for these receptors in its action. However, cafestol did not affect genes known to be upregulated by FXR in the liver of wild-type mice [132], indicating a more complex mechanisms of action.

### 12.3. Flavonoids

Flavonoids are polyphenolic compounds with several chemical differences that allow them to be classified into anthocyanidins, flavonols, flavanones, flavonols, flavones, and isoflavones. Administration of crude preparation of flavonoids from *Arabidopsis thaliana* seeds (15% w/w seeds for 4 weeks) significantly reduced DNA oxidative damage in the liver of Wistar rats compared to those fed the flavonoids-deprived seeds. Downregulation of genes associated with oxidative stress, Krebs cycle, electron transport and proteasome degradation was observed [133]. Other flavonoid such as quercetin at 0.1 or 0.5% in diet lowered the streptozotocin-(STZ-) induced increase in blood glucose levels and improved plasma insulin levels in mice. The highest dose suppressed the STZ-induced alteration of gene expression, particularly the elevation of cyclin-dependent kinase inhibitor p21 [134].

Dietary supplementation of isoflavones at 2 g/kg of either genistein or daidzein to rats showed that genistein was stronger than daidzein in targeting many hepatic genes that involved lipogenesis and carbohydrate metabolism [135]. Administration of soy yogurt downregulated the expression of the SREBP-1 gene and enzymes related to lipogenesis in the Sprague-Dawley rat liver, while expression of beta-oxidation-related genes was upregulated [136]. These results suggest that soy yogurt and its isoflavones are beneficial in preventing hepatic lipid accumulation in rats. 

### 12.4. Lignans

Lignans are polyphenolic compounds found in many plants. Compared to a lignan-free diet, a diet containing 0.2% sesamin, episesamin, and sesamolin modified the mRNA levels of many enzymes involved in hepatic fatty acid oxidation, proteins involved in the transport of fatty acids into hepatocytes and their organelles, and regulating hepatic concentrations of carnitine, CoA, and malonyl-CoA in male Sprague-Dawley rats. Lignans modified the gene expression of various proteins involved in hepatic lipogenesis, cholesterogenesis, and glucose metabolism. Episesamin and sesamolin induced greater changes than sesamin what is in agreement with their plasma and hepatic levels [137, 138]. In particular, sesamin significantly increased the expression of beta-oxidation-associated enzymes in peroxisomes and in mitochondria. Its ingestion also increased gene expression of acyl-CoA thioesterase very-long-chain acyl-CoA thioesterase and aldehyde dehydrogenase [139]. On the other hand, the transcription of the genes encoding the enzymes for fatty acid synthesis was decreased [140]. These results suggested that sesamin ingestion regulated the transcription levels of hepatic metabolizing enzymes for lipids and alcohol [141]. In this action, PPAR*α* plays a crucial role since mice lacking this receptor die when they are fed with sesame. However, the fact that a PPAR*α* ligand alone could not induce most of metabolizing enzymes indicates that there is an essential interaction among PPAR*α* and other xenobiotic nuclear receptors to induce the detoxification system [142]. Other lignans such as gomisin A recovered the carbon tetrachloride-induced rat liver damage and modified 255 upregulated and 230 downregulated genes. Increased gene expressions were related to cell cycle and suppression of the gene expression related in cell death [143].

### 12.5. Royal Jelly

Royal jelly is a honey bee secretion collected for human dietary supplement. Administration of isocaloric diets containing 5% royal jelly (RJ) modified the hepatic expression of 267 genes in mice with variations higher and lower than 1.8-fold or more. Decreased gene expression of squalene epoxidase, a key enzyme in cholesterol biosynthesis and sterol regulatory element-binding protein-1, and increased gene expression of low-density lipoprotein receptor may explain its hypocholesterolemic action [144]. Moreover, many genes involved in cell growth, signal transduction, energy metabolism, and transcription regulation were responsive to this diet. Among the 267 genes whose expression was altered by RJ, 60% showed no change or a reduced change in response to a diet containing 5% RJ stored at 40 degrees C for 7 days. In fact, this stored RJ diet contained little 57-kDa protein, identified as a possible freshness marker. Furthermore, the RJ diet did not influence the gene expression of cytochrome P450 enzymes and detoxifying enzymes, whereas the stored RJ diet increased the gene expression of glutathione S-transferase and glutathione peroxidase. Indeed, the RJ diet decreased the gene expression of cytochrome P450 4A14 (*Cyp4a14*), which catalyzes peroxidation of endogenous lipids that are associated with nonalcoholic steatohepatitis and alcoholic liver disease, while the stored RJ diet was not effective to decrease the gene expression of *Cyp4a14*. The results indicate that the efficacy of RJ decreased and the toxicity of RJ increased during storage at high temperature. According to these data, DNA microarray technology as an effective quality control procedure may be also applied to evaluate food safety [145].

### 12.6. Others

Transcriptomic analyses have been used to prove the effect of many other compounds. Traditional medicines such as *Salacia reticulata* stem upregulated catechol-o-methyltransferase and succinyl-CoA synthetase expressions in mice [146]. Coptis rhizoma displayed a unique antiproliferation pattern via p53 signaling, p53 activated, and DNA damage signaling pathways in HepG2 cells [147]. Chinese nutgall extract administered to male Kunming mice had a profound impact on expression of genes involved in metabolism, DNA binding and transcription, protein synthesis and modification, cell cytoskeleton and cell adhesion, cell cycle and differentiation, ion channels and transporters, signal transduction, immune response, and apoptosis [148]. Silymarin altered the transforming growth factor-beta-mediated pathways, which may represent antifibrotic effects. It also downregulated the expression levels of cytoskeleton organization genes and mitochondrion electron-transfer chain genes, such as cytochrome c oxidase *Cox6a2*, *Cox7a1*, and* Cox8b* genes [149]. Thus, they may be effective as functional foods.

Other active minor components provided through beverage such as teas, juices, or fortified foods have been studied. In this sense, catechin-rich green tea downregulated expression of genes for glucose-6-phosphatase and fatty acid synthase and upregulated expression of peroxisome proliferator activated receptor alpha in the rats, suggesting an antidiabetic activity [150]. An analogue, epigallocatechin-3-gallate as dietary supplement, influenced the antioxidative enzyme activities and their gene expressions in male Fischer 344 rats, suggesting that it may either function as an antioxidant by itself or regulate other bioprocesses, including energy metabolism, biosynthesis, and stress response [151]. Tomato and paprika beverages were also highly active in modifying gene expressions, both promoted glycogen accumulation and fatty acid oxidation [152]. Cocoa ingestion suppressed the expression of genes for enzymes involved in fatty acid synthesis and enhanced thermogenesis mechanisms in rat liver [153]. 8% peach or nectarine extracts had an effect on repair of various oxidative DNA lesions in livers from C57Bl/6J and this was related to an increase in the expression of endonuclease III-like Protein 1 [154]. Administration of the mushroom *Pleurotus ostreatus* to mice upregulated *Ctp1a* and *Fabp* genes which promoted lipid transport and beta-oxidation. The use of *Grifola frondosa* increased expression of genes involved in signal transduction of innate immunity via TLR3 and interferon and of those involved in virus resistance, such as *Mx1*, *Rsad2*, and *Oas1 *[155]. The extract from persimmon peel modified expression of insulin signaling pathway-related genes and increased insulin receptor beta tyrosine phosphorylation, improving insulin resistance in the liver of type 2 diabetic Goto-Kakizaki rats [156]. A final metabolite of panaxadiol ginsenosides, CK, shifted glucose metabolism from hepatic glucose production to hepatic glucose utilization in the liver and also improved insulin sensitivity [157]. Resveratrol decreased lipidogenesis and increased genes involved in the insulin signaling pathway and the glutathione metabolism in Wrn mutant mice, improving their hyperglycemia and insulin resistance [158]. Mangiferin, a compound in *Salacia reticulata*, downregulated the gluconeogenic fructose-1,6-bisphosphatase in liver of KK-Ay diabetic mice, decreasing fasting blood glucose levels [159]. In these mice, turmeric oleoresin ingestion upregulated the expression of genes related to glycolysis, beta-oxidation, and cholesterol metabolism in the liver, while expression of gluconeogenesis-related genes was downregulated. Turmeric oleoresin ingestion-induced expression of glycolysis-related genes and curcuminoids and turmeric essential oil acted synergistically to regulate the peroxisomal beta-oxidation-related gene expression induced by turmeric oleoresin ingestion. Therefore, the use of whole turmeric oleoresin is more effective than the use of either curcuminoids or the essential oil alone *in vivo* [160]. *Curcuma aromatica* oil (78 *µ*g/mL) was also found to decrease the expression of TIMP-2 and IL-6 in hepatic stellate cell line T6 and curcumol (1.6 *µ*g/mL) decreased TGF*β*1 and P450a [161]. In cultured hepatocytes and hepatoma cells, a clove extract reduced phosphoenolpyruvate carboxykinase and glucose 6-phosphatase gene expression acting as an insulin-mimetic agent [162]. In HepG2 and DU145 cells, fucoxanthin induced GADD45A, a cell cycle-related gene, together with a concomitant G_1_ arrest. siRNA against GADD45A partially suppressed the induction of G_1_ arrest by fucoxanthin [163]. As evidenced, these minor components exert important effects when present in extracts and in other cases when the molecule responsible is isolated and higher amount is required.

## 13. Alcohol

Although not a necessary dietary component, its intake may be important in populations and its gene changes as well. Surprisingly, with the keywords employed a low number of studies were found. Sake (15% alcohol) administration for 13 months induced liver damage in rats where 80 and 62 genes were up and downregulated, respectively. Decreased mitochondrial function and increased glycoproteins were the main changes [164]. In this animal, an alcohol-containing liquid diet (Lieber-DeCarli) for a shorter period of 14-15 weeks also induced significant transcriptional changes in genes involved in apoptosis and cell transport [165]. Using this technology, new mechanism of alcohol action is emerging.

## 14. Studies Dealing with Food Additives

Some food additives have been tested and found to induce changes in gene profiling in Sprague-Dawley rats. In this regard, butylated hydroxytoluene induced changes in the hepatic expression of 10 genes, including phase I (*Cyp2b1/2, Cyp3a9, Cyp2c6*) and phase II metabolism (*Gstmu2*). Curcumin altered the expression of 12 genes: three out of these were related to peroxisomes (phytanoyl-CoA dioxygenase, enoyl-CoA hydratase, *Cyp4a3*) suggesting that it is a weak peroxisome proliferator. Thiabendazole changed the expression of 12 genes, including *Cyp1a2*, *P53,* and genes associated with p53 such as *Gadd45alpha*, *Dn-7*, protein kinase C beta, and serum albumin. Propyl gallate changed the expression of eight genes [166].

The influence of 200 mg/kg butylated hydroxyanisole was tested in Nrf2-knockout and wildtype C57BL/6J mice. 493 genes, respectively, were identified as Nrf2 dependent and upregulated, and 824 genes, respectively, as Nrf2 dependent and downregulated. These genes can be categorized into ubiquitination/proteolysis, apoptosis/cell cycle, electron transport, detoxification, cell growth/differentiation, transcription factors/interacting partners, kinases and phosphatases, transport, biosynthesis/metabolism, RNA/protein processing and nuclear assembly, and DNA replication genes. Phase II detoxification/antioxidant genes as well as novel molecular target genes, including putative interacting partners of Nrf2 such as nuclear corepressors and coactivators, were also identified as Nrf2-dependent genes [167]. Food additives are important modifiers of transcriptome and need to be considered in future studies.

## 15. miRNA

The influence of dietary components has not only been studied on the codifying transcripts but also on the regulatory, particularly miRNA. In this aspect, the emergence of RNA sequencing has been crucial. Indeed, 517 miRNAs were identified in baboons: 490 identical to human and 27 novel. Eighteen miRNAs exhibited differential expression in response to a high-cholesterol, high-fat diet in high LDL-C baboons compared to 10 miRNAs in low LDL-C baboons. miRNAs expressed in high LDL-C baboons had significantly more gene targets than miRNAs expressed in low LDL-C responders [168]. Using the same technology, 150 miRNAs were found in mouse liver. Hepatic *miR-27b* levels were 3-fold upregulated in the liver of mice on a high-fat diet (42% calories from fat) and this miRNA regulated the expression of several key lipid-metabolism genes, including *Angptl3* and *Gpam* in Huh7 cells [169].

Other dietary manipulation such as Lieber-DeCarli and methionine-choline-deficient (MCD) diets leading to development of liver steatosis have been subjected to miRNA investigation as well. Both diets induced miRNA changes being more severe in animals receiving MCD diets. Of miRNAs that changed expression levels compared to corresponding controls, 5 were common in both diets, although with different response while miR-705 and miR-1224 were increased; *miR-182*, *miR-183*, and *miR-199a-3p* showed a different pattern: downregulated in Lieber-deCarli diet and upregulated with MCD diet. These data point out to a potential role for miRNA in liver steatosis and may discriminate between alcoholic and nonalcoholic origins [170]. Using a more dramatic dietary intervention as proposed by administration of a amino acid-defined and choline-deficient diet resulting in hepatocarcinogenesis, oncogenic *miR-155*, *miR-221/222,* and *miR-21 *were upregulated and the most abundant liver-specific *miR-122 was* downregulated, even at early stages of receiving this diet. Reduced expression of hepatic phosphatase and tensin homolog (*Pten*) and CCAAT/enhancer binding protein beta (*C/ebpbeta*), respective targets of *miR-21* and *miR-155*, were observed in C57BL/6J mice at early stages of steatosis. This diet also elevated DNA binding activity of nuclear factor kappa B that transactivates *miR-155* gene [171]. Overall, these miRNA may play an important role in liver pathology. 

Specific dietary components may also influence. Indeed, epigallocatechin gallate (EGCG) modified several miRNAs in HepG2 cells. One of the upregulated was *miR-16, *known to target the anti-apoptotic protein Bcl-2. EGCG treatment induced apoptosis and downregulated Bcl-2 in these cells. Transfection with anti-miR-16 inhibitor suppressed the miR-16 expression and counteracted the EGCG effects on Bcl-2 downregulation and also induction of apoptosis in cells. Thus, *miR-16* plays a role in the regulation of the biological activity of this compound [172]. Other compound such as furan, found in heated food items, also modified miRNA expression in Sprague Dawley rat liver [173]. Based on these observations, miRNA may play an important role in diet response.

## 16. Conclusion

This paper reviews studies dealing with hepatic high-throughput gene expression at the transcriptomic level after dietary manipulations. The intensity and nature of changes observed with such varied stimuli, including fasting/feeding, caloric restriction, dietary carbohydrate, cholesterol, fat, protein, bile acid, salt, vitamin and oligoelement contents, nature of fats or proteins, minor dietary components, alcohol, and food additives, evidence the availability of a potent technology and the sensitivity and metabolic flexibility of liver to adapt to these conditions. A profound elaboration of data is required to establish patterns of response in the today fragmented information puzzle. This will be a long road due to the lack of standardized arrays to systematically test the same genes in all studies, the different responses among strains and animals, the problems of analyzing data, and the emerging of new even more powerful technology such as RNA sequencing which may provide a new twist on the degree of information obtained. Undoubtedly, the analysis of miRNA will bring a new perspective to the field. Moreover, RNA is always an intermediate variable whose reflect on proteins may change, and in this way, it will be a valuable, easy-screening ally but always requiring further confirmation.

## Figures and Tables

**Table 1:** Nutritional status research subject of microarray analyses.

Condition	Model	Finding	References
Fasting	Mouse	Energy generation in early and glucose and glycogen synthesis in prolonged fasting	[35]
Fasting/feeding	Mouse	Modulation of PPAR*α*	[37]
	Rat	Increased abundance of protein in polysomes	[40]
Caloric restriction	Mouse, rat	Changes in stress response, xenobiotic metabolism, and lipid metabolism mediated by PPAR*α*	[41, 46]

**Table 2:** Influence of major nutrients according to microarray analyses.

Condition	Component	Model	Finding	References
Carbohydrates	Glucose	Mouse	↑ Oxidative stress	[49]
	Sucrose	Mouse	↑ *Pparg2 *	[50]
	Sweet corn	Mouse	↑ Cell proliferation	[51]
	Maple syrup	Rat	↓ Ammonium	[52]
	Fructooligosaccharide	Rat	↑ FXR	[54]

Amount of fat	Bolus of fat	Rat	↑*A2m, Slc13a5* and *Nrep *	[56]
	High fat diet	Mouse	↓*Cyp3a, Scd1 *	[57, 58]
			↑Inflammation	[59]
	High fat diet	Rat	↓ beta-oxidation	[60]

Nature of fat	MUFA, PUFA	Rat	↓ Lipogenesis	[61]
	n-3 PUFA	Mouse, rat	Modified cellular regulators	[62–64]
	CLA	Hamster, mouse, rat	Variable on the genetic background	[65–67]

Cholesterol	High level	Mouse	↓ CREP	[68]
	Modest level	Mouse	↑Inflammatory response	[69–72]

Protein	Soy versus casein	Rat	↓ Lipogenesis	[73, 74]
	Branched AA	Rat	↓ Ammonium, FA uptake	[75]
	Low methionine + choline	Mouse	↓ Oxidative stress	[76]
	Low methionine + choline + folate	Rat	Alteration of DNA methylation	[77]

AA: amino acids, CLA: conjugated linoleic acid; CREB: cAMP response element-binding protein; FA: fatty acid; MUFA: monounsaturated fatty acid; PUFA: polyunsaturated fatty acid.
